# Retraction: Zeng, Y.; Xiong, N.; Park, J.H.; Zheng, G. An Emergency-Adaptive Routing Scheme for Wireless Sensor Networks for Building Fire Hazard Monitoring. *Sensors* 2010, *10*, 6128–6148.

**DOI:** 10.3390/s110302898

**Published:** 2011-03-04

**Authors:** Shu-Kun Lin

**Affiliations:** MDPI AG, Postfach, CH-4005 Basel, Switzerland; Office: Kandererstrasse 25, 4057 Basel, Switzerland; E-Mail: lin@mdpi.com

It has been brought to our attention by a reader of *Sensors* that there existed arguments on both authorship and financial support for this article [1]. After confirming with all authors, we have determined that indeed this manuscript violates the generally accepted policies and ethics of scientific publishing. Consequently, the Editorial Team and the Publisher have determined that the original paper should be retracted and republished with the authorship and financial support fully disclosed. The paper has been republished as [2]. We apologize for any inconvenience this may cause to our readers.
